# Start Time End Time Integration (STETI): Method for Including Recent Data to Analyze Trends in Kidney Cancer Survival

**DOI:** 10.3390/healthcare13121451

**Published:** 2025-06-17

**Authors:** Thobani Chaduka, Daniel Berleant, Michael A. Bauer, Peng-Hung Tsai, Shi-Ming Tu

**Affiliations:** 1 Department of Information Science, University of Arkansas at Little Rock, 2801 S. University Ave., Little Rock, AR 72204, USA; tmchaduka@ualr.edu (T.C.); ptsai@ualr.edu (P.-H.T.); 2 College of Medicine, University of Arkansas for Medical Sciences, 4301 W. Markham St., Little Rock, AR 72205, USA; mbauer2@uams.edu (M.A.B.); stu@uams.edu (S.-M.T.)

**Keywords:** survival, cancer, kidney, treatment, trend, STETI

## Abstract

**Background/Objectives:** Accurately estimating survival times is critical for clinical decision-making, treatment evaluation, resource allocation, and other purposes. Yet data from relatively recent diagnosis cohorts is strongly affected by right censoring that biases average survival times downward. For example, 5-, 10-, or 20-year survival time averages are not available until 5, 10, or 20 years later, which may be in the future, thus presenting a challenge to obtain in the present. An approach to addressing this problem is described in this report. Here it is demonstrated for kidney cancer survival but could also be applied to survival questions for other types of cancer, other diseases, stage progression times, and similar problems in medicine and other fields in which there is a need for up-to-date analyses of survival improvement trends. **Methods**: This study introduces STETI, an approach to survival estimation that integrates information about survival times of diagnosis year cohorts with information about survival times of death year cohorts. By leveraging data from death year cohorts in addition to the more familiar diagnosis year cohorts, STETI incorporates recent survival data often excluded by traditional approaches due to right censoring, caused when the post-diagnosis time period of interest has not yet elapsed. Using data from SEER, we explain how the proposed approach integrates diagnosis year cohorts with the death year cohorts of recent years. We demonstrate that incorporating death year cohorts addresses an important source of right censorship that is inherent in diagnosis year cohorts from relatively recent years. This permits survival time trend analysis that accounts for recent improvements in survival time that would be difficult to account for using diagnosis year cohorts alone. We tested linear and exponential models to demonstrate the method’s ability to derive survival time trends using valuable data that would otherwise risk being left unused. **Conclusions**: Improved survival estimation can better support personalized treatment planning, healthcare benchmarking, and research into cancer subtypes as well as other domains. To this end, we introduce a hybrid analytical approach that addresses an important source of right censorship. Demonstrating it within the domain of kidney cancer is expected to help pave the way to other applications in oncology and beyond, and offers a case study of STETI, an approach to quantifying and projecting trends in survival time associated with therapeutic advancements.

## 1. Background

### 1.1. Epidemiology and Significance of Kidney Cancer

Kidney cancer ranks among the ten most common cancers, representing over 3% of reported cancer cases globally (Ferlay et al., 2013 [1]; Wong et al., 2017 [2]). While advances in diagnostics and treatment have improved patient outcomes, the disease presents a complex epidemiological profile with diverse subtypes. Renal cell carcinoma (RCC) predominates and accounts for over 90% of these cases (Naik et al., 2024 [3]). While the incidence of RCC is increasing (Padala et al., 2020 [4]), posing an important challenge, other types continue to present their own challenges in impact, diagnosis, and treatment. Thus, transitional cell carcinoma can occur in the kidneys (Tang et al., 2023 [5]), forming many of the non-RCC cases. Rarer subtypes also occur. Examples include medullary renal carcinoma. Highly treatable but only when correctly diagnosed, one case went viral with millions of views (Chubbyemu, 2021 [6]). Another rare subtype, Wilms tumor, can occur even before birth (Bechara et al., 2024 [7]). This underscores the heterogeneous nature of kidney cancer across age groups and histological subtypes, and the associated distinct diagnostic and therapeutic challenges.

### 1.2. Importance of Accurate Survival Estimation

Estimating survival time contributes to kidney disease prognosis and guides patients, their families, and clinicians in making decisions about treatment and future planning (Kardaun, 1991 [8]). Many patients want reliable survival estimates (Hagerty et al., 2005 [9]), as it empowers them to understand their journeys as well as helping them with emotional and financial preparedness. From a clinical perspective, survival predictions help in both designing personalized treatment protocols and optimizing follow-up schedules, thus improving overall care. At an institutional level, accurate survival data are needed for benchmarking treatment center performance (Wong et al., 2017 [2]), thus helping to drive improvements in clinical practices.

Additionally, survival estimates can contribute to the healthcare system overall, such as in identifying cost-effective protocols for screening, treatment, and follow-up, ultimately impacting healthcare policies, guidelines, and costs.

Survival statistics and trends also play a significant role in research. They enable evaluating new treatments and can help direct resources toward subtypes with the greatest potential for treatment improvement. Yet as treatments improve, traditional methods of survival analysis often fail to fully account for the trend in improvement, potentially leading to outdated estimates. This limitation highlights the need for methodologies that adapt to the dynamics of improvement in cancer treatment by using all available data in their analyses, even (or especially) data that is recent enough to be challenging to leverage owing to the right censoring of survival times inherent in recent diagnosis cohorts.

### 1.3. Current Methods and Their Limitations

Existing survival estimation techniques have notable constraints. The Kaplan–Meier method is widely used in survival analysis. It estimates survival probability over time based on observed survival data, adjusting for patients that are lost to follow-up and thus whose data is censored and hence incomplete. However, the Kaplan–Meier method, while addressing the right censored data problem, does not directly address trends of change in survival over time.

Cox proportional hazards models are widely used for analyzing covariate effects. They can analyze the relationship between survival time and predictor variables (age, stage, biomarkers, etc.) and provide estimates of hazard ratios which can facilitate calculating survival probabilities. While a time variable like diagnosis date can take the role of a predictor variable, relatively recent diagnosis year cohorts present a challenge when the complete data on their associated survival statistics is right censored as it is not yet available.

General approaches feature wide applicability. However, disease-specific methods can address the characteristics of particular diseases. Such models often integrate multiple sources of evidence, thus providing a comparatively comprehensive evaluation. For instance, nomograms (Iasonos et al., 2008 [10]; Kou et al., 2021 [11]) are widely recognized for modeling specific cancers and are particularly useful in generating individualized predictions. They can calculate the probability of survival for a specified period, such as five years, by integrating multiple sources of evidence, including clinical, pathological, and molecular data, providing both estimates and error bounds. However, traditional nomograms, such as the ASSURE model for renal cell carcinoma (Correa et al., 2021 [12]), fail to incorporate recent advancements in therapeutic approaches like targeted therapies and immunotherapy. Thus, nomograms can become dated, not accounting for improvement trends in treatment, hence leading to out-of-date forecasts for new diagnoses. This is a limitation in conditions, like kidney cancer, with significant trends of survival improvement.

Survival prediction for kidney cancer, like other forms of cancer and other illnesses, has increasingly been explored using data mining and machine learning. These approaches leverage large datasets, derived from such sources as clinical records and cancer registries like SEER and others. Numerous such techniques have emerged in this era of advanced data analysis. Random forests, for instance, have been extensively studied since even before the recent surge in neural network research (Shi et al., 2005 [13]) and continue to be investigated (Ranjan et al., 2022 [14]). Decision trees boast an even longer history, with their application predating the 21st century (François et al., 1999 [15]) with continued interest today (Souza-Silva et al., 2024 [16]). Neural network models, first proposed in 1958 (Rosenblatt [17]), have in recent years surged to the forefront of artificial intelligence research and practice and show significant potential for survival prediction in kidney and other cancers (Song et al., 2024 [18]). However, a key limitation of neural networks is their current inability to provide clear explanations for the results they generate. This challenge remains a critical focus of ongoing research (Yenduri et al., 2024 [19]). Addressing this issue will be essential for realizing the full potential of neural-network-based predictions in kidney cancer and beyond.

Addressing the limitations of all these methods when they rely on historical data that may no longer reflect the realities of current medical advancements is an ongoing need. Analyses should provide up-to-date forecasts that account for trends of improvement in treatments, ensuring more accurate and relevant predictions. Bayesian approaches form one approach founded on Bayes’ theorem, which explains how posterior probabilities may be updated from priors. This approach enables combining new evidence with the old when probability is involved. There is potential for applying this approach to merging older diagnosis year cohort evidence with newer death-year-based evidence to update the probabilities associated with different survival times. For example, Bartoš et al. (2022 [20]) demonstrated the integration of older survival data with newer data and how to apply this to continuous updating with new evidence as it becomes available.

When evidence and desired conclusions are not explicitly probabilistic, other methods are needed, either alone or hybridized with Bayesian or other probabilistic approaches. We have not addressed the potential of hybridization here, instead focusing on developing a foundation, the STETI approach, that could be hybridized and extended in other ways in future work.

### 1.4. The Problem and Its Resolution

If the problem was merely to identify the trend of improvement in survival over time and extrapolate from older data to the present and future times, curve fitting would be a simple solution. However, extrapolating from a fitted curve is only the first part of the problem. The second is to use the newer data to assist. This is important because deriving improvement curve trajectories works better with more data and—because trend characteristics can vary over time (e.g., Mazzoleni et al., 2021 [21]; Muggeo, 2008 [22])—newer data has even more value than older data. Yet new data is not complete enough to support strong conclusions about N-year survival until N years have elapsed, at which time additional new data will exist with the same problem. For example, calculating metrics such as the cause-specific 10-year average survival time requires using diagnoses from over 10 years ago. This is a problem because the old data may tell an outdated story that does not account well for the survival benefits accruing from recent treatment improvements.

One approach to accounting for trends of survival improvement using the more challenging, newer data is period analysis. This method was first adapted to the medical field by Brenner and Gefeller (1997 [23]). Their solution to using the newer data about relatively recent diagnosis years for which survival data is not yet available for the entire time period of interest is to impute values for unavailable years (Brenner et al., 2004 [24]). Their imputation process may be typified by an example, from which the reader may generalize. Suppose we need survival numbers for each year of a 10-year period for diagnoses made in 2020, but the survival data available only goes through 2024. To impute the survival figure for 2025, use the 2024 figure for 2019 diagnoses. Similarly, to impute the 2026 survival for 2020 diagnoses, use the 2024 number for 2018 diagnoses; for the 2027 survival, use the 2024 number for 2017 diagnoses, and so on. However, some inaccuracy in the analysis remains because of the necessity to rely on imputing future data using historical data, a process which does not fully adjust imputed values for any trend that might exist in treatment effectiveness. Such adjustments could be estimated and used, but these would nevertheless be uncertain. They report results that are more accurate than those obtained by merely ignoring data from recent diagnosis years, thus not accounting for recent improvements in outcomes.

The method we describe here, Start Time End Time Integration (STETI), incorporates newer data from diagnosis years that mark the start of time periods that have not ended yet. To avoid imputing data that does not yet exist, the method shifts the focus from start (diagnosis) year cohorts to end year cohorts. A curve modeling the trend of survival time improvement is fitted to the end-year-based data. This curve is different but arithmetically interconvertible with the curve that would be fitted to the diagnosis-year-based survival data if that data existed. However, since the two curves are interconvertible, the end-date-based curve is then converted to a diagnosis date curve. This estimates survival from the diagnosis date as desired, while incorporating recent survival data not used in a more conventional analysis.

STETI, because it relies on regression, can be used when the amount of data is not statistically large enough to conceal stochastic variation (noise). However, this does lead to the unavoidable limitation that there may be insufficient data to do this reliably. Another inherent limitation is that fitting a curve means assuming the mathematical formula of the curve is a valid model of change in the effectiveness of treatment over time. Thus, the method, while providing distinctive advantages, has limitations as well.

### 1.5. Objective of the Study

This study targets improved survival estimation with a novel method, STETI (Start Time End Time Integration), that does trend analysis that incorporates newer survival data that reflects outcomes of more advanced treatment methods. This data category is challenging to leverage because it is mixed in with right censored data points of patients who are still alive. For example, the goal of determining the 20-year average survival time of a diagnosis cohort from 10 years ago is challenging because survival times are available only for patients who survived 10 years or less, while the survival times of those who will survive 11–20 years are currently right censored. This biases the average downward, a problem that becomes progressively more severe for progressively more recent diagnosis cohorts.

To address this challenge, the method leverages death year cohorts instead of only diagnosis year cohorts. Death year cohorts by definition do not contain right censored records, so this is a key step to using some (though not all) relatively recent data that would otherwise be biased due to the right censorship inherent in recent diagnosis year cohorts. When modeling trends of increases in survival time—driven by advancements in treatment—we wish to account for the dynamics of improvement in cancer care enabled by the sequence of new treatments introduced over time, even when these improvements are relatively recent. This approach reduces over-reliance on older data that can underestimate survival outcomes by not accounting for the newest treatments.

This article describes the development, implementation, and validation of this approach, highlighting its application to kidney cancer. However, it is a general technique with applicability to other cancer types, subtypes, and broader medical and other contexts. When there is a trend of improvement in survival times, STETI is intended as an approach to utilizing up-to-date survival data that would otherwise be challenging to use, thus enabling improved survival estimation.

## 2. Method

STETI starts with a model of improvement in average survival time as treatment changes over time. Like the base approach, it is keyed to start date (e.g., diagnosis year) as the independent variable and estimates average survival time as a function of start year. However, unlike the base approach, it also uses recent data not used in the base approach by employing the following process.

Steps of the STETI modeling process:

(1) Specify a type of model for average survival time as a function of the start year cohort. (We will assume start year is the diagnosis year in the following, for ease of exposition, but it could be defined as a stage transition or other starting time.)
(2) Transform the function of the diagnosis year algebraically into a function of the death year.
(3) Regress parameters in the function of the death year to make it fit the data. The data consists of an average survival time associated with each death year cohort.
(4) Algebraically transform the death year curve, with its parameters now specified, back into a diagnosis year curve.
(5) Use the new diagnosis year curve for predictions and estimates as desired.

We next expand on each of these steps, providing details to more fully explain how the method works.

### 2.1. Specify a Type of Model Estimating Average Survival Time as a Function of the Diagnosis Year Cohort

Treatment extends patient average survival time for most serious diseases. Because medicine, like other fields in which science and technology play roles, tends toward improvement over time, expected survival times often increase over time. We might choose to model this increase as a linear (i.e., constant rate of) improvement over time, or instead, as an exponential function of time, a logistic function, or some other type of curve. Once fitted to the historical survival data, different such models will often give similar predictions for present and near-future diagnoses since the fitting process will cause them all to attempt to follow the underlying trend behind the historical data.

**Figure 1:**
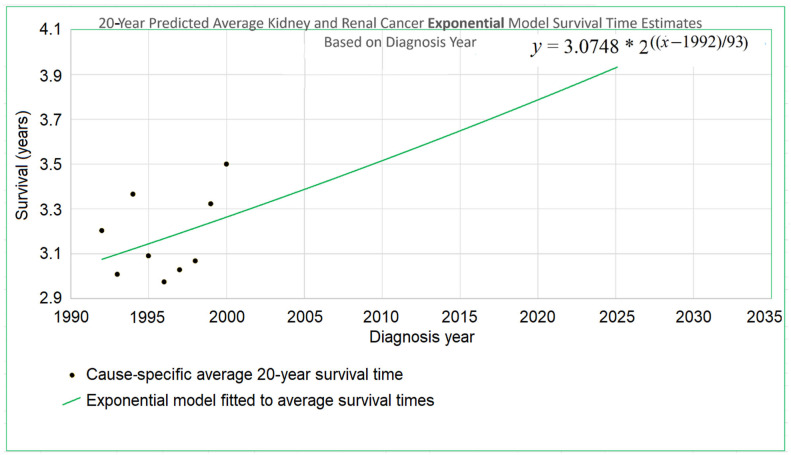
Twenty-year average survival times for kidney cancer deaths are shown for the diagnosis years 1992–2000. An exponential curve fitted to the data using Excel’s trendline functionality extrapolates predictions for times after 2000; the data points for which are unavailable. This is due to the 20-year wait for complete survival data, highlighting the limitations of the base approach (a full analysis is given later).

An example appears in Figure 1. The 20-year average survival times for kidney cancer are shown for the years 1992 through 2000. The 20-year average survival time for a diagnosis year cohort is defined as the mean survival time for patients who do not survive more than 20 calendar years after the year of diagnosis. These averages are not available for recent diagnosis years because the 20-year average survival time is not available until 20 years after the diagnosis year. The exponential curve ${y=3.0748*2}^{(x-1992)/93}$ was fitted to the data points with the help of the Excel trend curve functionality. Note that the most recent data point is for year 2000 diagnoses, because data was unavailable past 2020. Year 2000 is a long time ago to be the most recent diagnosis year cohort to analyze, especially as so many patients have been diagnosed more recently than that and have benefited from relatively recent advances in treatment technology. While a significant amount of survival data for individual patients is available for more recent diagnosis years, that data is a challenge to use to compute a 20-year average survival time because not all of the needed 20 years of data exist yet. Simply waiting 20 years until full data is available we will call the base approach.

The method reported here, STETI, addresses this challenge by transforming the diagnosis-year-based analysis into a death-year-based analysis. The 20-year average survival time for a death year cohort is defined as the mean survival time for patients in a death year cohort who were diagnosed up to 20 calendar years prior to the death year. Average survival times for death year cohorts are available for years that are more recent than 2000; up to 2020 for the dataset we used. Thus, for example, the 2020 death year cohort includes survival times for diagnoses in 2000, 2001, …, 2020. This includes diagnoses in years like 2008 for which the 20-year average survival time will not be available until the data on 2028 deaths is available. Thus, diagnoses in 2008 would not be used in the base method even if they appear in the 2020 death year cohort and hence are used in STETI. The transformation process is explained next.

### 2.2. Transforming the Function of the Diagnosis Year Algebraically into a Function of the Death Year

As noted, a basic cohort analysis of survival time based on diagnosis year would involve following a cohort for 5, 10, or 20 years after the diagnosis year to be able to calculate a statistic like 5-, 10-, or 20-year average survival time. Relatively new data is left unused. This makes the conclusions of the base method out of date for the many cancers for which advances in treatment over time lead to ever-improving outcomes. Even if a delay of 5 years was tolerable, the 20-year survival analysis results are likely to misjudge the outcomes for current patients who have experienced improved treatment protocols adopted within the last 20 years. A model of survival as a function of death year would enable using many data records too recent to be used by the base method.

We next derive a death-year-based model from a linear diagnosis-year-based model. The reader may if desired skip the following subsection without loss of continuity. Analogous derivations for exponential and logistic diagnosis year models are provided in Appendix A.

#### Deriving a Death Year Survival Model from a Linear Diagnosis Year Model

To begin, consider a typical general equation for a linear model (i.e., a straight line on an *xy*-plane):

(1) y=mx+b

where *y* is the height of the line for any value of *x* on the horizontal axis, and *b* is the *y*-intercept, or height of the line where it crosses the *y*-axis (sometimes but not necessarily where *x* = 0). In the present context, we may restate the equation as follows:

Estimated average survival time for a given diagnosis year              = (Rate of improvement in average survival time per year) ∗ (Diagnosis year − 1990)     + (Estimated average survival time for diagnoses in 1990)(2)

where the *y*-axis is placed so as to cross the x-axis at the year 1990 for convenience and because we do not wish to suggest that a model of modern cancer survival would be applicable in year 0, thousands of years ago. Equation (2) modifies Equation (1) by specifying a particular application and also by placing the *y*-axis so it crosses the *x*-axis at year 1990 instead of year 0.

Restating Equation (2) more concisely gives

(3) $$survival(t_{begin})\;=slope\;*\;(t_{begin}-1990)\;+\;survival\;(1990)$$

and restating again we get

(4) $$S_{t_{b}}\left(t_{b}\right)=M*\left(t_{b}-1990\right)+S_{t_{b}}(1990)$$

indicating survival as a function of begin time, $S_{t_{b}}(\;)$, given the begin time parameter *t_b_*, where *M* is the slope.

We wish to transform Equation (4), which estimates average survival time given the time of diagnosis, into an equivalent model estimating average survival time given the time of death. This requires moving *t_b_* and $S_{t_{b}}\left(t_{b}\right)$ out of Equation (4) and, instead, using *t_e_* and $S_{t_{e}}\left(t_{e}\right),$ where *t_e_* is an end time (time of death in the present context), and $S_{t_{e}}$( ) is a function estimating average survival time given an end time.

Observe that (end time) = (begin time) + (survival time), that is,

(5) $${t_{b}+S}_{t_{b}}\left(t_{b}\right)=t_{e}$$

Similarly,

(6) $$t_{e}-S_{t_{e}}\left(t_{e}\right)=t_{b}$$

Therefore,

(7) $$S_{t_{b}}\left(t_{b}\right)=S_{t_{e}}\left(t_{e}\right)$$

Removing *t_b_* and $S_{t_{b}}\left(t_{b}\right)$ from Equation (4) using Equations (6) and (7),

$$\begin{array}{cc} S_{t_{e}}\left(t_{e}\right) & =M\left(t_{e}-S_{t_{e}}\left(t_{e}\right)-1990\right)+S_{t_{b}}\left(1990\right)\\ & =Mt_{e}-MS_{t_{e}}\left(t_{e}\right)-1990M+S_{t_{b}}\left(1990\right)\\ \mathrm{Rearranging}\;\mathrm{terms},\;(M+1)S_{t_{e}}\left(t_{e}\right) & =M\left(t_{e}-1990\right)+S_{t_{b}}\left(1990\right) \end{array}$$

and so

(8) $$S_{t_{e}}\left(t_{e}\right)=\frac{M}{M+1}t_{e}+\frac{S_{t_{b}}\left(1990\right)-1990M}{M+1}$$

Note that Equation (8) also happens to be linear, with the survival time function $S_{t_{e}}\left(t_{e}\right)$ having the slope *M*/(*M* + 1) and a value for survival time for the year at which the curve crosses the *y*-axis, which is *x* = *t_e_* = 1990, of ${[S}_{t_{b}}\left(1990\right)\;-\;1990M]/(M+1).$

### 2.3. Regressing the Death Year Curve to the Data

If treatment did not improve over time, the base method would be reasonable for estimating average survival time because the older data it relies on would be substantially equivalent to newer data. However, since the treatment of kidney cancer is improving, the base analysis is unreliable as it leaves out the very relevant recent data.

**Figure 2:**
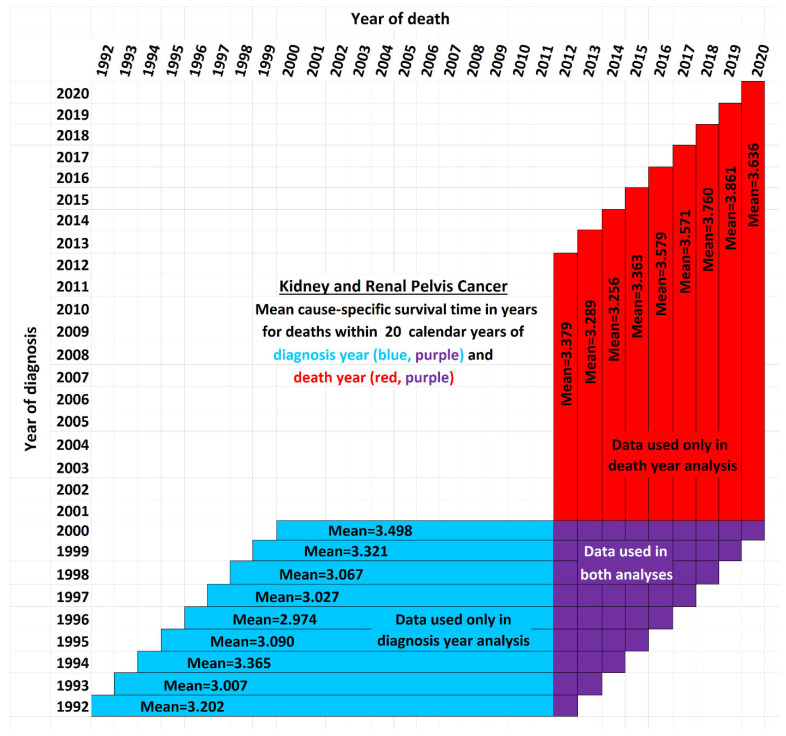
Twenty-year survival time cohorts. The blue and purple horizontal bars represent diagnosis year cohorts, while the red and purple bars represent death year cohorts. Red cells in the top right area of the chart show relatively recent survival data included in the red and purple vertical 20-year cohort bars. They contain relatively recent information used in STETI but not in the base method. Blue cells in the horizontal blue and purple 20-year cohort bars contain historical data used in the base method.

Figure 2 illustrates the issue for the case of 20-year survival times, or those patients surviving up to 20 years after the year of diagnosis. It shows a schematic view of the new data used by STETI but not the base method (red cells). Blue and purple cells are used by the base method, and by STETI too because STETI can integrate the results of the base method as part of its analysis. Although the time period of each vertical red and purple bar matches the time period of a horizontal blue and purple bar, they differ in a critical way: each horizontal bar represents a diagnosis year cohort, while each vertical bar represents a death year cohort. Thus, a vertical bar contains red cells with more recent diagnoses than are found in any blue cell. By including the red cells in its analyses, STETI handles the problem of patients in diagnosis year cohorts which are too recent to have 20 more years of follow-up (for a 20-year average survival time analysis), as long as they are in a death year cohort for which data exists for each of its preceding 20 diagnosis years. Thus, a patient survival time can be analyzed if it is (i) in a diagnosis year cohort with 20 more years of follow-up death data, and/or (ii) in a death year cohort with 20 years of preceding diagnosis data. This enables including many data records (the red cells) whose diagnosis years are too recent to have 20 years of follow-up.

The white cells fall into three categories: (i) those above the diagonal, which are empty because a diagnosis year is not normally after a death year; (ii) those in the lower right corner, which represent survival times more than 20 years before or after a cohort year, and (iii) the remainder, found in the empty area of the “V” between the blue and red regions, which contain valid data that is nevertheless unused either by the base method or by STETI. Those cells are not in any 20-year cohort defined by a diagnosis year (because data was not yet available for the full 20 years of follow-up as required), nor are they in any 20-year cohort defined by a death year (because reliable data was not available for the full 20 preceding years as required). Perhaps a way could be devised to make use of these cells. Note that this chart reflects that treatment technology is improving: each red bar except one has a higher mean survival time than its corresponding blue bar (*p* < 0.02).

By including the consideration of death year cohorts, the problem of recent diagnosis year cohorts being biased by only containing shorter survival times is addressed: those diagnosis year cohorts are not analyzed directly, but many of the patients in those cohorts are in death year cohorts that are analyzed.

On the other hand, records of patients who were lost to follow-up or who are still surviving are censored as usual, since no survival times are available for those records.

The models for the linear, exponential, and logistic trends of improvement in survival times have parameters that need to be specified in order to describe the specific trend curves that could be designed to fit actual data and plotted on a graph. In linear modeling, Equation (4), we specify a slope parameter, *M*, and a *y*-axis intercept value, $S_{t_{b}}\left(1990\right)$. These parameters describe linear models of how survival improves with increasing diagnosis year. The models of linear survival improvement with increasing death year, Equation (8), have the same two parameters. Note, however, that although Equation (8) is linear, its slope is not *M* but $\frac{M}{M+1}$, and its *y*-intercept is not $S_{t_{b}}\left(1990\right)$ but $\frac{S_{t_{b}}\left(1990\right)-1990M}{M+1}$. Nevertheless, Equation (8) can be fully defined by specifying values for *M* and $S_{t_{b}}\left(1990\right)$, which are to be determined based on the data from the diagnosis year and death year cohorts.

The linear, exponential, and logistic models all have a rate parameter (respectively, slope, doubling time, and maximum steepness). The models also each have a *y*-intercept parameter (the curve height for the year 1990 in the linear case, and for the other two, see Appendix A). The rate and *y*-intercept parameters are each discussed individually in the next two subsections.

#### 2.3.1. Determining the y-Intercept Parameter

Since diagnosis year modeling is well-known and established, we will use it for data analysis up to the most recent year *t_r_* for which the base method works. Years more recent than that require death year modeling. Year *t_r_* and its survival time estimate provided by the diagnosis-year-based modeling also provide a starting point for the trajectory of a survival curve predicted by a death-year-based model. For instance, in Figure 1, this point is at the year 2000, the time of the last average survival time data point available to diagnosis-year-based models, and the height of the point is 3.262, the height of the curve for the year 2000. We therefore set the height in 2000 at 3.262 for exponential modeling in Figure 1.

A few questions about this method are considered next.

FAQ:

Q: Why not optimize the fit of the death year curve to the data by regressing it on both the *y*-intercept and steepness parameters, instead of setting the *y*-intercept and regressing just the steepness parameter?

A: This is a modeling decision. An argument for setting the *y*-intercept using the diagnosis year analysis is that diagnosis year analysis uses more of the historical data (blue cells in Figure 2), thus reducing the stochastic noise problem that bedevils smaller datasets. An argument for using only death year data to determine both the *y*-intercept and steepness parameters is that newer data may better reflect the current underlying trend if it has changed over time. Modeling decisions are often judgment calls that depend on problem specifics and the modeler’s experience.

Q: How can a true shift in models be distinguished from shifts arising due to such factors as noise in the data, apparently random fluctuations in the model parameters over time, and discontinuous changes due to discontinuous shifts in treatment methods where these changes may or may not counterbalance each other over time?

A: This is a difficult question that surrounds the problem of trend analysis. See, e.g., Farmer and Lafond (2016) [25].

Q: Cannot multiple modeling approaches be used instead of focusing on just one model? Then their results could be combined to provide an average prediction and/or a range or set of predictions.

A: Yes, as for example, the spaghetti diagrams used for hurricane path prediction, showing the different paths predicted by the numerous different weather simulation models (Track the Tropics, 2024 [26]).

#### 2.3.2. Determine the Steepness Parameter

**Figure 3:**
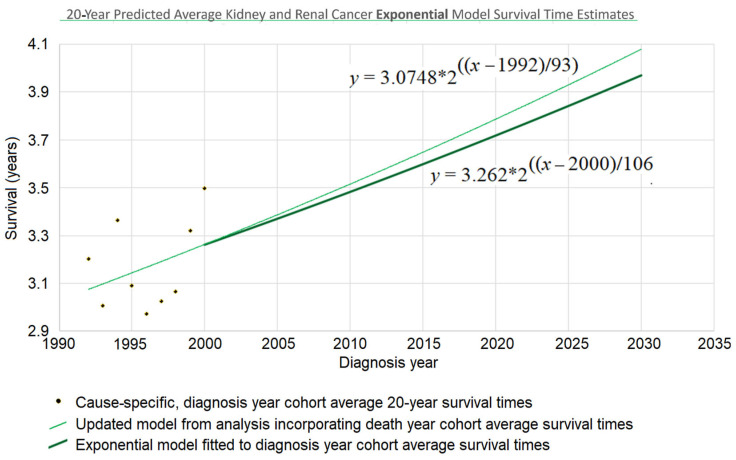
A diagnosis-year-cohort-based trend curve and an updated trend curve using STETI model the trajectory of improvement in cause-specific 20-year average survival time for kidney and renal cancers (full analysis appears later).

Once the *y*-intercept parameter has been specified, the remaining parameter to determine is the steepness. To find the steepness, regress the steepness parameter in the function $S_{t_{e}}\left(t_{e}\right)$ that predicts survival time from the end date *t_e_* (for “end time”) to the data points that associate survival times with death years. For the case of a linear model, that means regressing Equation (8). For the exponential model of Figure 1 above, the mathematical details are provided in Appendix A.4. The result of this process was *d* = 106 (see Figure 3), and the web page which calculated that (Berleant 2024 [27]) is online.

### 2.4. Transforming the Death Year Curve Back into an Updated Diagnosis Year Curve

Survival time as a function of death year, $S_{t_{e}}\left(t_{e}\right)$, has now been regressed based on Figure 1 and the red and purple data of Figure 2. This death year curve can be transformed back into the diagnosis-year-based curve (which gives survival time as a function of diagnosis year) by plugging the *y*-intercept and steepness into the standard linear (Equation (4)) or exponential (Figure 3 heavy curve) equation. This will update the curve of Figure 1 to account for the red data of Figure 2.

Continuing the example accordingly, we have (i) the *y*-intercept parameter $S_{t_{b}}\left(2000\right)=3.2623$, and (ii) the doubling time parameter *d* = 106. Plugging them into the exponential equation of Figure 3 (thin curve) modifies it to give

$$S_{t_{b}}\left(t_{b}\right)=3.26232411*2^{(t_{b}-2000)/106}$$

which is graphed in bold in Figure 3. The new curve was developed under the assumption that the curve provided by the base method (using diagnosis year cohorts only) is valid up to the year 2000. Thus, we graph the new curve starting from the year 2000. (It should be noted that, while this assumption is plausible, other assumptions may be plausible as well. See the spaghetti diagram discussion below in this section.)

**Understanding the discrepancy between the base method and STETI**. Several factors may contribute to the differences between the predictions of the two approaches.

- *Noisy data.* Random and seemingly random fluctuations may affect the data due to stochastic, temporary, unmeasured, and/or unmodeled factors. For instance, external influences such as pandemics, economic cycles, etc., can introduce variability that the model does not account for. This unpredictability highlights the limits of modeling complex phenomena, because models inherently simplify an endlessly complex reality.
- *Changes in model parameters over time.* Changes in the reality being modeled can occur, changing the relationships among model variables. In such cases, a single model might not effectively represent the dynamics over an extended period. A regression model may then need to be piecewise, modeling different parts of the time period differently. The Chow test is commonly used to handle such break points. Other tests such as the CUSUM test (Cumulative Sum of Residuals) and the Bai–Perron test can also be useful in dealing with model shifts over time. However, the application of such methods is unclear in cases like the 20-year kidney and renal cancer survival example, where the base analysis approach is used on earlier data while more recent data uses the new approach.
- *Short-term variation with reversion to the mean.* Sometimes shifts in a trajectory over time can be short-term variations within the context of a more consistent long-term trend. For example, a model of long-term economic growth might need to accommodate shorter-term recessions within an overarching trend rather than as evidence against such a trend. Similarly, models of technological advancement may show long-term trends which are composed of short-term segments caused by specific changes in the technology (e.g., Park 2017 [28]). Thus, it is important to consider that changes in a trajectory may be merely temporary excursions from the longer-term trend rather than fundamental shifts.
- *Spaghetti diagrams, ensembles, and cones of uncertainty.* It would be convenient if there were a single best model, but an ensemble combining multiple models may more effectively represent the spectrum of possible outcomes. Spaghetti diagrams exemplify this approach by providing a visual representation of the outcome paths. This approach is commonly used, for example, in meteorology, where spaghetti diagrams are widely utilized to show the range of trajectories of hurricanes predicted by different weather models (Belles 2024 [29]). Figure 3 depicts a simple 2-trajectory spaghetti diagram, the trajectories forming a small ensemble used to depict a cone of uncertainty.

## 3. Results

Using the new approach, we analyzed kidney and renal cancer data from SEER 12 (National Cancer Institute 2025 [30]), querying for data on patients who were diagnosed and passed away from kidney and renal pelvis cancer throughout the years 1992 to 2020. SEER provides de-identified patient records, including the year of diagnosis and death, for a substantial portion of U.S. cancer cases. These records come from regions with rigorous reporting standards that help ensure high data quality and encompass various cancer types.

Average survival times were calculated for yearly diagnosis cohorts and for yearly death cohorts over three periods of observation: 5, 10, and 20 years. Since each cohort required survival data for a full 5, 10, or 20 years to be analyzed, this constrained which cohorts were usable. Figure 2 exemplifies the details using the 20-year case. For each time span, both the exponential and linear models were applied. The results are next.

### 3.1. Five-Year Average Survival Times

**Figure 4:**
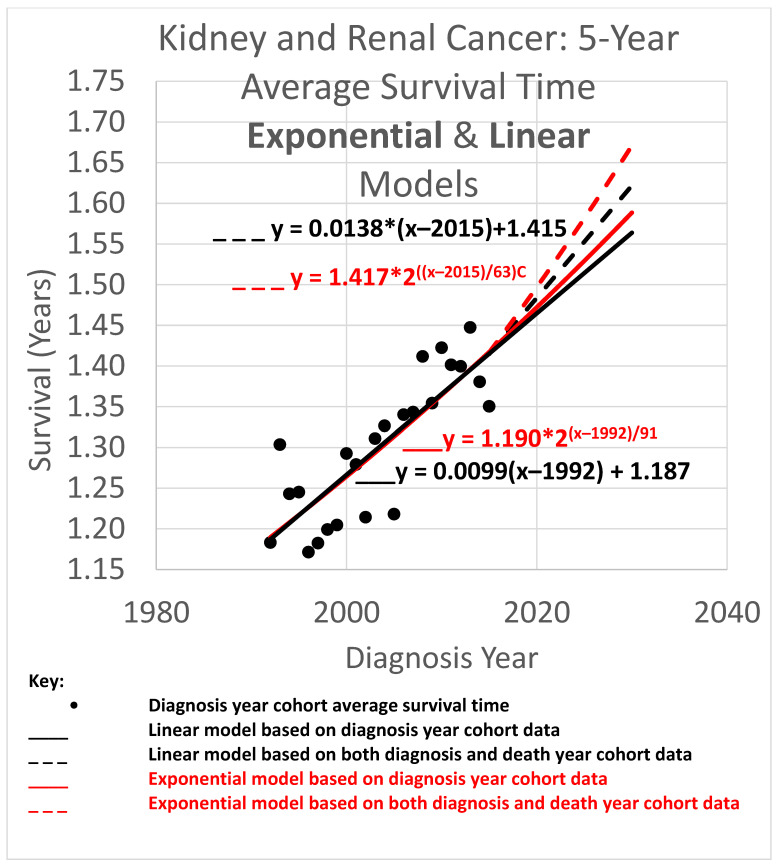
Kidney and renal cancer data from SEER were used to calculate cause-specific 5-year average survival time curves. These modeled the trend of improvement in 5-year average survival time. Linear and exponential models (solid curves) were fitted to diagnosis year cohort averages (dots), and finally, updated curves (dashed) were computed that additionally account for death year cohort data.

The 5-year average survival time is the mean survival time of patients who survived as long as 5 calendar years after their diagnosis year. Five-year average survival time trend analyses are shown in Figure 4. The data obtained covered the years 1992 through 2020. Since the curves shown extend to the year 2030, they are necessarily extrapolations after 2020. In the graph, a height represents survival time in years. The horizontal axis gives diagnosis year values. Since each data point represents a 5-year average survival time for a diagnosis year cohort, they only go up to 2015, since for the diagnosis years of 2016 and beyond, survival data for the following 5 years, required to calculate the 5-year average survival time, was not available.

The root mean squared error (RMSE) was 0.027 for the linear model and 0.028 for the exponential model; the Akaike information criterion (AIC) was −171.0 (linear) and −170.4 (exponential), giving exp(Δ/2) = 0.75, which indicates no significant preference. The long doubling times in the exponential models (63 and 91 years) cause the exponential curves to look relatively straight and linear over the moderate time periods shown, although the solid curves are long enough to show the slight curvature of the red exponential as compared to the black linear curves. The linear and exponential dashed curves project 5-year average survival time for the 2025 diagnosis cohort (or for a patient in that cohort for whom a customized analysis is unavailable). For the linear model, the projection for diagnosis cohort year 2025 was 1.553 years. Comparing the projections of model variations of different slopes showed a range of [1.547, 1.565] years for slope variations giving AIC calculations of exp(Δ/2) > 0.5 (the most likely variations). For the exponential model, the projection was 1.582 years. Comparing the projections of model variations with different doubling times showed a range of [1.570, 1.589] years for doubling time variations giving AIC calculations of exp(Δ/2) > 0.5 (the most likely variations).

Since neither the linear nor exponential model is preferred, we averaged their projections to give (rounding the 2nd decimal place) the 2025 diagnosis year cohort an estimate of 1.57 years.

### 3.2. Ten-Year Average Survival Times

**Figure 5:**
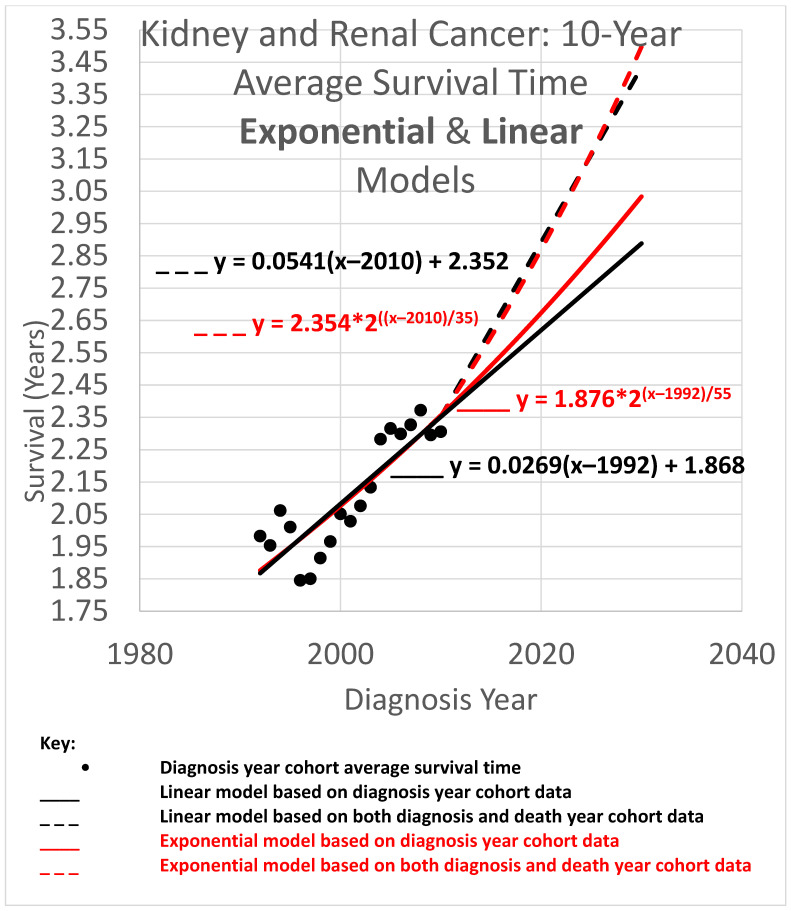
Trends of improvement in cause-specific 10-year average survival time.

The 10-year average survival time is the mean survival time of patients who survived up to 10 calendar years after their diagnosis year. Ten-year average survival time trend analyses are shown in Figure 5. Since each data point represents an average for a diagnosis year cohort, they only go up to 2010, since for diagnosis years 2011 and beyond, survival data for the following 10 years required to calculate the 10-year average survival time, was not yet available.

The root mean squared error (RMSE) was 0.098 for the linear model and 0.075 for the exponential model; the Akaike information criterion (AIC) was −86.1 (linear) and −96.7 (exponential), giving exp(Δ/2) = 0.005, leaning toward the exponential model as a descriptor of the data. The long doubling times in the exponential models (35 and 55 years) cause the exponential curves to look relatively straight and linear over the moderate time periods shown, although the red exponential curves do show visible curvature relative to the corresponding black linear curves. The linear and exponential dashed curves project 10-year average survival time for the 2025 diagnosis cohort. For the linear model, the projection was 3.253 years. Comparing the projections of the model variations of different slopes showed a range of [3.074, 3.464] years for model variations with an AIC calculation of exp(Δ/2) > 0.5 (the most likely variations). For the exponential model, the projection was 3.168 years. Comparing the projections of model variations with different doubling times showed a range of [2.964, 3.382] years for model variations with AIC calculations of exp(Δ/2) > 0.5.

Since AIC analysis prefers the exponential model, we can use the equation of the exponential dashed curve to project the 10-year average survival time for the 2025 diagnosis cohort, or a patient in that cohort without a customized analysis (rounding the 2nd decimal place), of 3.17 years.

### 3.3. Twenty-Year Average Survival Times

**Figure 6:**
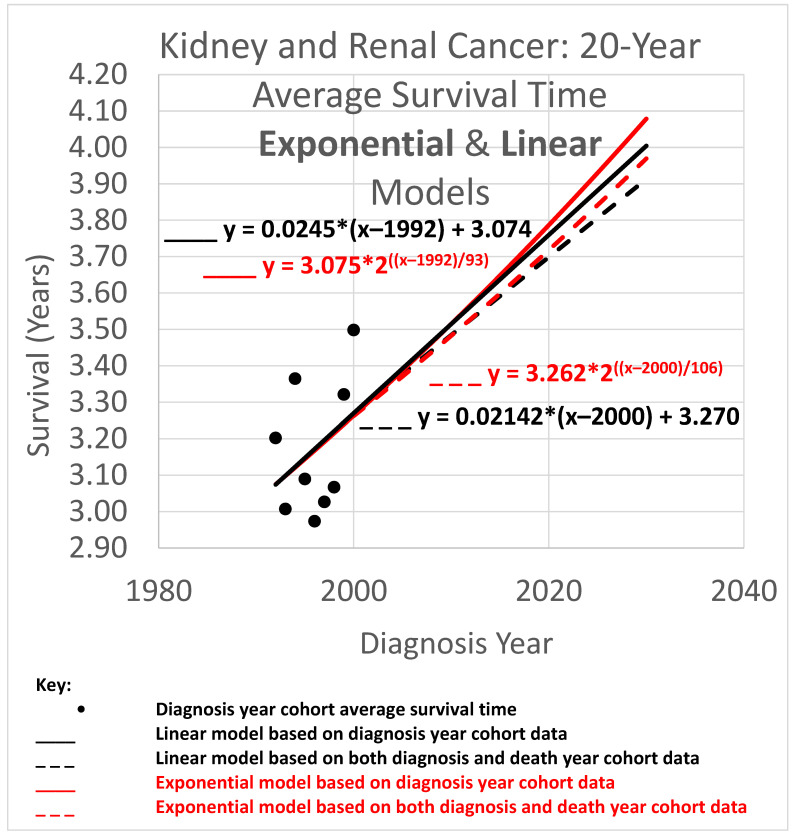
Trends of improvement in cause-specific 20-year average survival time over observation periods of 20 years after diagnosis year.

The 20-year average survival time is the mean survival time of patients who survived up to 20 calendar years after their diagnosis year. Twenty-year survival time trend analyses are shown in Figure 6. Since each data point represents an average for a diagnosis year cohort, they only go up to 2000, since for diagnosis years 2001 and beyond, survival data for the following 20 years, required to calculate the 20-year average survival time, was not yet available.

The root mean squared error (RMSE) was 0.043 for the linear model and also 0.043 for the exponential model; the Akaike information criterion (AIC) was −54.5 (linear) and −54.8 (exponential), giving exp(Δ/2) = 0.86, indicating little or no exp(Δ/2) preference for one model over the other as a descriptor of the data. The long doubling times in the exponential models (93 and 106 years) cause the exponential curves to look relatively straight and linear over the moderate time periods shown, although a close look shows that the red exponential curves do show visible curvature relative to the corresponding black linear curves. The linear and exponential dashed curves project 20-year average survival times. For the 2025 diagnosis cohort, or a patient in that cohort without a customized analysis, the linear model projection was 3.806 years. Comparing the projections of model variations of different slopes showed a range of [3.667, 3.940] years for model variations with an AIC calculation of exp(Δ/2) > 0.5 (the most likely variations). For the exponential model, the projection was 3.842 years, with projections of model variations with different doubling times showing a range of [3.699, 3.973] years for model variations with AIC calculations of exp(Δ/2) > 0.5 (the most likely variations).

Since AIC analysis gives the linear and exponential models essentially equal preference, we can use the mean of the models’ projections (3.806 and 3.842) to estimate average survival time for the 2025 diagnosis cohort, or a patient in that cohort without a customized analysis (rounding the 2nd decimal place), of 3.82 years.

### 3.4. Average Survival Times over All Observation Periods

The results of Figure 4, Figure 5 and Figure 6 show qualitative commonalities for 5-, 10-, and 20-year average survival times. A welcome result of all the models, base and STETI, is that average survival times are increasing. Thus, using survival estimates from bygone years will underestimate survival for newly diagnosed renal carcinoma patients, unless the trend of improvement is accounted for. This is why both the base and STETI methods provided more optimistic average survival times than would be obtained by methods that use older data while failing to account for trends of improvement. If there was no trend of improvement, then relying on estimates from older data would be expected to show survival times as high as would be found by accounting for newer data. Trend estimates that use STETI to leverage both older and more recent data (dashed curves) vary from trend estimates that rely only on the older data and thus ignore the more recent data that is more challenging to use.

There is no particular reason to expect STETI results to provide faster- or slower-rising curves than base analyses. The analyses above showed modestly more optimistic projections for the two scenarios of 5- and 10-year survival, but slightly less optimistic (but still upward-trending) projections for the 20-year survival scenario. Two out of three has no statistical significance. Still, it may be useful to note some other possible reasons for discrepancies between the base and STETI curves as well as among the 5-, 10-, and 20-year scenarios. One is that error bounds indicate ranges of uncertainty around projections that could alleviate (or enhance) apparent discrepancies. We used the AIC-based measure of exp(Δ/2) to assess the difference between two models or model variations. The ranges given used the criterion exp(Δ/2) > 0.5, which is rather restrictive on the ranges. Models that fall outside those ranges are certainly plausible, and ranges that are wide and inclusive enough to exclude only significantly lower likelihood model variations, such as would be obtained, e.g., from a criterion like exp(Δ/2) > 0.05, would yield wider error bounds than those given in the results for each scenario. Another factor in causing the various differences in the projections of the various models and variations is changes in the rate of increase in average survival times caused by new treatments, external socio-economic changes, and other exogenous influences. Newer data might reflect the influence of changing conditions. An additional factor that could lead to different rates of improvement across the 5-, 10-, and 20-year scenarios is that treatment improvements might have differential impacts among these groups. A further consideration is that there is a period of years which produce data for the 5- and 10-year scenarios but do not provide data that can be used by the 20-year scenario. For example, the year 2007 provides neither diagnosis year cohort data (since 20 years have not yet elapsed as of this writing) nor death year cohort data (since data was not available for 20 years prior to 2007). This is illustrated in more detail in Figure 2: the V-shaped region of white cells are not in an available 20-year stretch of data that would permit them to be part of the analysis.

The recent data used by STETI should lead to adjustments in survival estimates because of this additional data, but not to very large differences in estimates given reasonably well-behaved data and no revolutions in treatment.

That is what we see in the analyses above.

### 3.5. Retrospective Prediction Experiments

**Figure 7:**
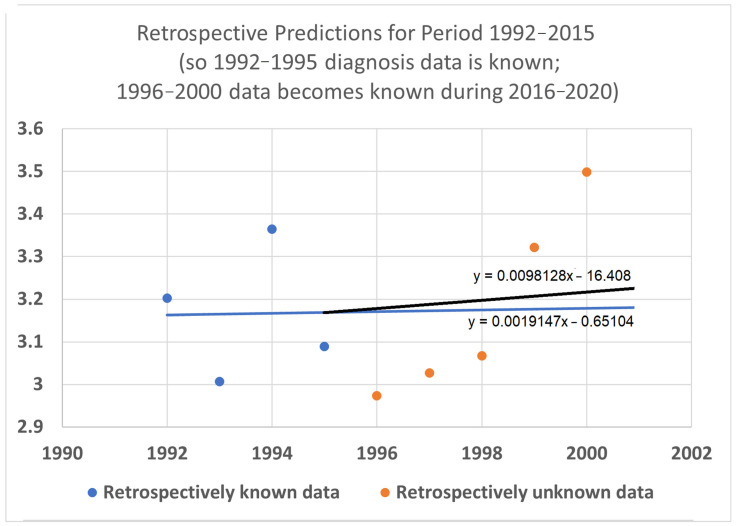
Given 20-year average survival times for diagnosis year cohorts 1992, 1993, 1994, and 1995 (blue dots), and death year cohorts 2012, 2013, 2014, and 2015, predict the 20-year average survival times for held-out diagnosis year cohorts 1996–2000 (orange dots). The blue line is the linear regression to the blue dots (RMSE = 0.197, AIC = −12.22). The black line uses STETI to also include the death year cohort data (RMSE = 0.188, AIC = −14.71). The black STETI line predicts the held-out orange data better than the base case blue line regression, but not by much (RMSE 0.188 vs. 0.197) and, comparing AIC values, exp(Δ/2) = e^(−14.71−12.22)/2^ = 0.29 indicating a small (not statistically significant) preference for the STETI result. Therefore, we stress-tested the methods on a more limited dataset. The result is shown in Figure 8.

To help validate the STETI approach, tests were designed using the 20-year average survival time scenario with linear modeling. Earlier subsets of the data were used to generate predictions of improvement in survival time, and the held-out later subsets were compared to the predictions. In the first test (Figure 7), data only from 1992–2015 was used, instead of the 1992–2020 data collected and used for the results described above. This meant looking at four diagnosis year cohorts for which 20-year survival averages could be determined: the 1992 cohort (followed through 2012), and the 1993, 1994, and 1995 (followed through 2013, 2014, and 2015, respectively) cohorts. It also included four death year cohorts: 2012 deaths from diagnoses back to 1992, and the 2013, 2014, and 2015 (followed back to diagnoses in 1993, 1994, and 1995, respectively) cohorts. Since the original full dataset included diagnosis cohorts up to 2000, we could check how well STETI and the base method (regression on diagnosis year cohort data) predicted 20-year average survival times for diagnosis cohorts 1996 through 2000, using the held-out actual 20-year average survival times for those cohorts. The result is shown in Figure 7.

In this test, only three (instead of four) diagnosis year cohorts were used, representing data only from 1992 to 2014. The 1995 diagnosis year cohort was no longer used because determining its 20-year average survival time would require data from 2015, which was held out in this test. We then compared how well STETI predicted the held-out orange dots compared to the base case of regression on the blue dots.

The black STETI line predicted the held-out orange data better (RMSE 0.22 vs. 0.41) and, comparing AIC values, exp(Δ/2) = e^(−16.34−6.76)/2^ = 0.00028 indicates a statistically significant preference for the STETI result. This is perhaps not too surprising because the predictive ability of a linear regression to three data points will often be inordinately affected by stochastic or unaccounted-for variation in the three values. In contrast, STETI also considers three additional data points from 20 years later, and while those data points are partially dependent on the blue dots (via the purple cells in Figure 2), they also reflect independent data as well (red cells in Figure 2).

We then further restricted the input data by limiting the base and STETI analyses to the period 1992–2013 (Figure 9). This permitted only the two diagnosis year cohorts of 1992 and 1993 to be used to calculate 20-year average survival times and only two death year cohorts, 2012 and 2013, to additionally be used by STETI.

The blue base case line is regressed to the two blue dots, so it is the line that goes through both points. The black STETI line uses the blue dots and, in addition, the 20-year average survival times for death year cohorts 2012 and 2013. Two somewhat noisy data points form a poor basis for a predictive regression line, so the blue line consequently predicts the orange held-out data poorly. The STETI line, however, provides a plausible prediction of the orange data.

As a final test, we shrunk the data to use for prediction to just one diagnosis year cohort (2012), and its one associated death year cohort (2012). The result appears in Figure 10.

From 20-year average survival times for diagnosis year cohort 1992 (blue dot) and death year cohort 2012, STETI provided a forecast of the held-out diagnosis year survival averages for 1993–2000 (orange dots). The base case of using only one diagnosis year cohort cannot be regressed at all because one data point does not support fitting a slope. The black STETI line uses two data points, the 2012 diagnosis year cohort 20-year average survival time, and the 2012 death year cohort 20-year average survival time. This is enough to regress a line to, giving the reasonable result shown.

## 4. Discussion

### 4.1. Long-Term Trends in Treatment Are Composed of Multiple Short-Term Advances

Any longer term history of advancement in a technological domain will contain specific advances that were discovered, diffused into wider use, and eventually ceased to contribute directly to further advancement by achieving full market penetration and/or becoming replaced by newer and better advances. These individual advances each generally have logistic or other S-curve short-term characteristics that become components of an overarching long-term advancement process (Kucharavy and De Guio, 2011 [31]).

Considering kidney and renal cancer treatment as a technological domain in which overall advancement results from multiple specific advances, a list of specific modern drug advances is shown in Table 1.

With the long-term trajectory of treatment improvement composed of the effects of multiple specific short-term improvements, it is natural to conjecture that the long-term trajectory would have a stepwise appearance, with steps corresponding to individual improvements in treatment. However, an improvement generally exerts an influence that gradually rises, plateaus, and perhaps then falls (Figure 11). The effects of each improvement thus become smeared with the effects of others, smoothing any tendency toward a stepwise trajectory.

### 4.2. Comparing Methods, Models, and Results

The development of STETI and the results lead to a number of observations about both its contributions and its limitations.

*Average survival times are increasing*. A heartening aspect of the results is that all analyses, both linear and exponential, base analyses and STETI, and each time period tested, gave trajectories of increasing average survival time. Treatment is improving. As a result, failing to account for the historical and current trend of continuing improvement in treatment effectiveness will risk underestimating survival times for current patients. Thus, incorporating trend analysis in survival estimates is important, highlighting the need for advancing trend analyses and trend analysis techniques.

*STETI addresses a significant shortcoming.* Right censoring presents a challenge to using recent survival data, because survival time data from recently diagnosed patients cannot include the longer survival times of patients who survive past the present. This tends to make survival averages for recent diagnosis year cohorts too low. Period analysis addresses this problem by imputing the most applicable historical data that is available. STETI avoids data imputation, instead addressing this problem by shifting attention to death year cohorts, which do not have this particular problem. Thus, STETI can make use of relatively newer data that is not available to the base method (illustrated in Figure 2). On the other hand, STETI assumes a linear, exponential, or potentially other trend model whereas period analysis does not. Perhaps one day a way will be devised that combines the advantages of both methods.

**Figure 8:**
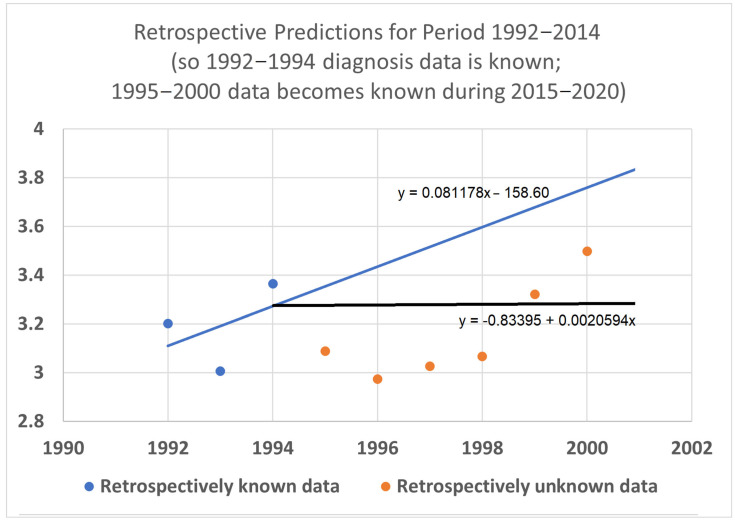
The 20-year average survival times for diagnosis year cohorts 1992, 1993, and 1994 (blue dots), and death year cohorts 2012, 2013, and 2014, predict the 20-year average survival times for diagnosis year cohorts 1995–2000 (orange dots). The blue line is the linear regression to the blue dots (RMSE = 0.44, AIC = −6.76). The black line uses STETI to also include the death year cohort data (RMSE = 0.22, AIC = −16.34).

**Figure 9:**
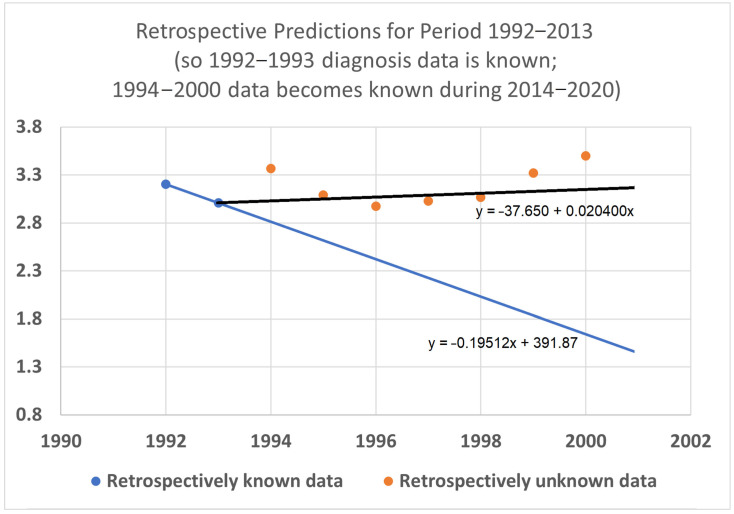
Given 20-year average survival times for diagnosis year cohorts 1992 and 1993 only (blue dots), and death year cohorts 2012 and 2013, predict the diagnosis year survival averages for 1994–2000 (orange dots). The blue line is the linear regression to the blue dots, and it missed the cluster of orange data that it putatively predicts (RMSE = 1.080). The black line uses STETI to also include the death year cohort data (RMSE = 0.20). It manages to hit the cluster of orange dots, indicating plausible predictive performance.

**Figure 10:**
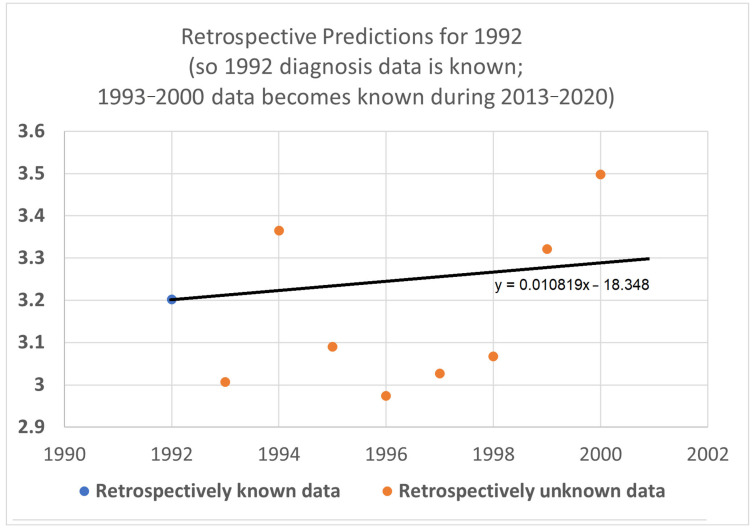
The STETI prediction line is shown. It is anchored by the one data point available from a diagnosis year cohort. It then proceeds through the cloud of orange held-out data that we wish to predict (RMSE = 0.19), its slope determined by the 20-year average survival time of the 2012 death year cohort.

**Figure 11:**
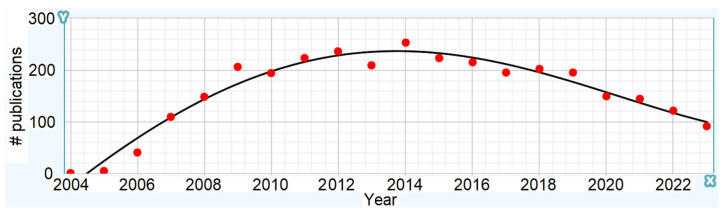
Number of publications per year on Sunitinib for the treatment of renal carcinoma. Sunitinib (Sutent) was approved by the FDA for the treatment of renal cancer at a specific point in time, 26 January 2006 (U.S. Food and Drug Administration, 2006 [32]). Yet its effects on renal cancer treatment, as indicated by the number of scholarly articles published about it per year, have been spread out over many years and indeed still continue (Google Scholar, 2024 [45]). (Graphing tool credit: https://mycurvefit.com, Beta version, accessed 1 May 2025).

The STETI approach described here uses more data than the base method alone. It leverages the base method to help determine the trajectory by setting the *y*-intercept parameter based on the diagnosis year cohort analysis, while also using additional recent death year cohort data to set the steepness parameter. The validation tests help illustrate the value of this. Figure 8, Figure 9 and Figure 10 all indicate a considerably better predictive performance by STETI compared to the base method, which performs poorly due to the small number of data points that it has to base its analyses on, producing results that are inordinately affected by stochasticity in the data.

*The right censoring problem is only partially addressed.* STETI addresses an important type of right censoring, in that it enables the use of data from recent diagnosis cohorts whose time horizon has not yet expired and thus would downward bias their average survival times if not carefully handled by shifting attention to death year cohorts instead. However, records of patients without recorded survival times, such as patients lost to follow-up and patients whose survival continues through to the present, are also right censored, and STETI does not attempt to handle those forms of right censoring.

*The difference in results across the two methods is often not dramatic.* The difference between the trajectories of the base method and STETI for near-term estimates is relatively modest in many cases. For example, Figure 4, Figure 5 and Figure 6 illustrate differences in average survival time predictions across the two methods; for diagnosis year 2025, it ranges from a few weeks to a few months. This relative concordance of predictions helps validate the foundations of both methods, without overshadowing STETI’s methodological advantages: using more and newer data is a plus, and survival time is important, so even modest differences are worth getting as accurately as possible. However, in limited data situations, differences can be greater and STETI has the advantage (Figure 8, Figure 9 and Figure 10).

*Linear vs. exponential models.* A comparison between the linear and exponential models whose trajectories are shown in Figure 4, Figure 5 and Figure 6 reveals that these models produce similar results. Modeling the rate of advancement in specific technologies as exponential is a common default choice in the technology foresight field with the rationale that the evolving state of a technology is likely to support a relatively constant percentage of improvement per year (Magee, 2016 [46]), which is an exponential rate of increase. The two best-known examples are probably Moore’s law for computer chips and the “Carlson curve” of the cost of DNA sequencing per base pair. For the present domain, average survival times must eventually approach an upper limit defined by the observation period (5, 10, or 20 years), suggesting that a logistic or other S-curve model might ultimately prove most appropriate for very long-term projections. However, the logistic curve is effectively exponential initially, transitioning to near linear in the regions around its inflection point. Another consideration is that exponential and other curved phenomena, when viewed over a short enough time span, become effectively linear. This is the case here, due to the relatively long doubling times that STETI found for the exponential models in all cases, which ranged from a low of 35 to a high of 106 years. Thus, the linear and exponential models gave generally consistent results in the experiments herein. This is quantified by the AIC exp(Δ/2) comparisons, which showed no consistent pattern of preference for either type of model. This concordance is unsurprising as both are tuned by the *y*-intercept and steepness to fit the same data. Unless the primary interest is in long-term extrapolation, an inherently speculative endeavor anyway, the question of what is the “right” model is not an overriding issue. Instead, by considering both simultaneously, they can potentially provide input to an uncertainty quantification analysis using spaghetti diagrams and cones of uncertainty (next).

*Ensembles, spaghetti diagrams, and cones of uncertainty.* Each set of results in Figure 4, Figure 5 and Figure 6 shows multiple trajectories, forming an ensemble of curves that led to spaghetti diagrams with diverging tendencies forming a cone of uncertainty. The paths forming the spaghetti diagram and its enclosing cone of uncertainty communicates uncertainty that increases over time using a range of representative outcomes. Rather than viewing different results from different methods as representing an unresolved conflict, it may instead be useful to acknowledge that all models are imperfect representations of reality. This motivates running multiple models as an ensemble. The differing predictions can then be usefully interpreted as revealing the inherent uncertainty in the prediction analysis. This perspective highlights the power of ensemble modeling to convey uncertainty and offer a richer representation of potential survival trajectories.

By integrating the outputs of multiple models, the ensemble framework can enhance understanding of the uncertainties in predictions. The cone of uncertainty also serves as a basic reminder of the limits of predictability in the behavior of complex systems.

### 4.3. Why Identify Trends of Improvement in Kidney Cancer Treatment?

The findings presented here have implications for both clinical practice and research methodology. For clinicians, the results suggest using survival estimates that incorporate the effects of recent treatment advances to obtain more reliable estimates. For researchers, trend findings that incorporate newer data and are thus more up to date give more timely projections of the likely magnitude of the benefits of future treatments, providing expectations that can help drive the discoveries required to achieve those benefits (Mulay, 2022 [47]).

## 5. Conclusions and Future Work

The work presented here provides a starting point for addressing its limitations as well as building on it with a range of applications and extensions.

*Further development of STETI.* Although STETI incorporates data not used by the base method, there is other data not used by either method. The white cells in Figure 2 between the arms of the red and blue V are each associated with a diagnosis year, death year, and number of patients, providing valid data not used in either the blue and purple base analysis or the red and purple additional data used in the STETI analysis. Further research might find a way to use these cells so as not to leave that data unused.

Another path of potential improvement is in handling modeling assumptions. While this study explored exponential and linear models of increasing average survival times, alternative models, such as logistic or other sigmoidal (S-curve) models, also warrant investigation. Moreover, the underlying drivers that are being modeled may shift over time, leading to the need for alternative modeling that can capture such shifts. A more flexible and diverse modeling toolbox would permit a richer characterization of long-term dynamics.

To avoid commitment to a particular model by imposing a linear, exponential, or other model onto the data, why not combine the results of different models by recognizing their different predictions as a form of uncertainty to be quantified? This could be achieved using ensembles, spaghetti diagrams, and cones of uncertainty to represent the variability across the results of different models. This would provide better visualizations of the uncertainties related to model choice, leading to fuller pictures of what can be concluded about relevant survival trends and their extrapolations.

Although STETI makes modeling assumptions, period analysis does not. Yet period analysis imputes data while STETI does not. Is it possible to combine the advantages of both methods into one? This question needs to be investigated further.

Because Kaplan–Meier survival functions are well established, it would be useful to modify STETI for application to Kaplan–Meier survival functions. When survival metrics improve over time, K–M survival functions change accordingly. To adapt STETI to this context, sets of K–M survival profiles of cohorts associated with different ranges of diagnosis time periods could be analyzed to quantitatively determine how metrics like median survival time or survival time at specific percentiles evolve over time. The K–M method is notable for its handling of right censored data. Yet in STETI, we have noted earlier that using death year cohorts does not consider patients who are lost to follow-up, and neither does using diagnosis year cohort patients known to have survived for less than the period of interest (such as 5, 10, or 20 years). However, these patients lost to follow-up might introduce bias. For example, patients who survived longer might be more likely to be lost to follow-up, biasing average survival times downward. This further motivates extending STETI by applying it within the K–M context. This would continue to advance the ability to deal with sources of right censorship.

*Simulation studies.* By offering the chance to exercise the method under simulated and thus controlled data conditions, properties of the method can be explored in a directed way. In contrast, relying on real data ensures applicability to real domain problems but can impede investigation of internal aspects of the method itself, like sensitivity and robustness to noise, performance across varying sizes and durations of datasets, sensitivity to bias caused by different sources of censoring, and assessing identification of predefined survival trends that are defined by the simulated data.

*Extension to new domains.* Future work is needed to apply STETI to other cancer types and subtypes. A broad cancer category like kidney cancer includes subtypes with differences in their treatment protocols and survival expectations, as well as their rate of increase in survival time due to improvements in treatment. Such subtypes could be defined genetically, for example, and this is expected to become more prevalent as genetic sequencing and profiling advances. The approach can be applied to other categories of cancers, such as myeloma and lung cancer (Chaduka, 2024 [48]). It can also be applied to subtypes of these and other cancers for which treatments are improving. We are currently investigating myeloma subtypes and have obtained preliminary results for the CD-2 genetic subtype. Similarly, survival after diagnosis is generalizable to survival after transition to a particular stage of cancer, to time in a particular stage, and so on. Additionally, there are various other databases available, many maintained by other countries around the world. Extending STETI analyses to these would help establish its applicability and also generalize its results, since despite the fact that different regions often have distinctive characteristics, improvements in treatment tend to cross national borders. Other serious diseases besides cancer could be analyzed similarly. The method also holds promise for application in non-medical domains such as engineering, where manufactured artifacts may exhibit increases in product life as their associated technologies advance (e.g., Howell et al., 2019 [49]).

*Benchmarking against alternative methods*. A benchmarking study comparing approaches is needed. Comprehensive comparisons of alternative methods make valuable contributions in many fields. Survival analysis would benefit from a new benchmarking study which would permit a comparison between STETI, period analysis, AI techniques like random forests and neural networks, and others. Such an effort would constitute a distinct project of significant effort. All reasonable methods will typically give reasonable results and thus might be expected to usually produce results that differ, but not greatly. Yet, more reliable results have value even if they are only incrementally different than other results, just as incremental advances are valuable in medical treatment and so many other endeavors. The problem then is verifying when one method gives preferable survival projections to another method. For this, a careful benchmarking study is needed because ad hoc or cherry-picked comparisons could be excessively affected by stochastic and other effects that can hide or confound conclusions from such comparisons.

Together, these directions highlight the potential to refine and extend the methodology, enhancing its applicability and impact across medical and other domains.

### Concluding Note

This study focuses on the critical problem of adapting survival estimation methodologies to account for the dynamic improvement over time in treatment outcomes. By introducing an end-date-based analysis method, we provide an approach, STETI, that addresses existing limitations in leveraging recent survival data, thus showing a path to more dependable survival predictions. We demonstrated the approach on kidney cancer, but its applicability is expected to extend to other cancer types, cancer subtypes, other diseases, and even non-medical domains. This approach to survival analysis advances the handling of right censored data and has the potential to help inform patients, their doctors, and research on treatment advances and research directions.

## Figures and Tables

**Table 1:** Some specific recent advances in kidney cancer treatment.

Drug	Approved	Reference
Sunitinib	2006	(U.S. Food and Drug Administration, 2006 [32])
Temsirolimus	2007	(U.S. Food and Drug Administration. 2007 [33])
Pazopanib	2009	(U.S. Food and Drug Administration, 2009 [34])
Zortress (everolimus)	2010	(Drugs.com, 2010 [35])
Axitinib	2012	(Tyler, 2012 [36])
Cabozantinib	2016	(U.S. Food and Drug Administration, 2016a [37])
Everolimus + Lenvatinib	2016	(U.S. Food and Drug Administration, 2016b [38])
Sunitinib adjuvant	2017	(U.S. Food and Drug Administration, 2017 [39])
Nivolumab + ipilimumab	2018	(U.S. Food and Drug Administration, 2018 [40])
Pembrolizumab + axitinib	2019	(Lane, 2019 [41])
Pembrolizumab + Lenvatinib adjuvant	2021	(U.S. Food and Drug Administration, 2021a [42])
Nivolumab + cabozantinib	2021	(U.S. Food and Drug Administration, 2021b [43])
Belzutifan	2023	(Drugs.com, 2023 [44])

## Data Availability

Data used in this study was obtained from SEER, https://seer.cancer.gov/registries/terms.html (accessed prior to 31 December 2022). It was tabulated for use in the research into tables provided in the appendices of Chaduka (2024). The software for providing the results illustrated in Figure 4, Figure 5 and Figure 6 is available as web pages containing embedded JavaScript for making the calculations upon request to the corresponding author.

## Abbreviations

The following abbreviations are used in this manuscript:

SEER:	Surveillance, Epidemiology, and End Results Program of the U.S. National Cancer Institute

## Appendix A

Below are the STETI derivations for the exponential and logistic models. They parallel the derivation for the linear model presented in the main text.

### Appendix A.1. Deriving a Death Year Survival Model from an Exponential Diagnosis Year Model

Transforming an exponential model of how survival time increases with advancing diagnosis year into a model of how it increases with advancing death year parallels the process for linear models.

One form of the general exponential equation is

(A1) $$y=cb^{x/d}$$

where *c* is the *y*-intercept or the height of the curve (e.g., the predicted survival time) where it crosses the *y*-axis. This is where the exponent is zero, in this case where *x* = 0. The base *b* and time constant *d* in Equation (A1) are redundant in that an exponential curve defined by setting *b* arbitrarily and then adjusting *d* as desired can alternatively be specified by setting *d* arbitrarily and adjusting *b*. We will use *b* = 2 because then increasing the exponent by 1 causes *y* to double. Thus *b* = 2 exposes the “doubling time” property frequently discussed in the technology foresight literature (e.g., Bias et al., 2014 [50]), with *d* being the doubling time.

For the present context, we restate Equation (A1) as

(A2) $$S_{t_{b}}\left(t_{b}\right)=S_{t_{b}}(2000)*2^{(t_{b}-2000)/d}$$

indicating survival as a function of begin time, with the *y*-axis shifted to cross the *x*-axis (here named the *t_b_*-axis) at the year 2000. We used 2000 here, and 1990 in the linear model description above, highlighting that the year chosen is arbitrary.

We wish to convert Equation (A2), which is a model estimating survival time from time of diagnosis *t_b_*, into an equivalent model estimating survival time from time of death. This requires replacing *t_b_* and $S_{t_{b}}\left(t_{b}\right)$ in Equation (A2) with *t_e_*, the end time or time of death, and $S_{t_{e}}\left(t_{e}\right)$ where $S_{t_{e}}$ is a function estimating survival time from end time.

As in the linear model scenario, we need to replace *t_b_* and $S_{t_{b}}\left(t_{b}\right)$ with *t_e_* and $S_{t_{e}}\left(t_{e}\right)$, using Equations (6) and (7) again. We restate them here for convenience as Equations (A3) and (A4).

(A3) $$t_{e}-S_{t_{e}}\left(t_{e}\right)=t_{b}$$

(A4) $$S_{t_{b}}\left(t_{b}\right)=S_{t_{e}}\left(t_{e}\right)$$

Applying Equations (A3) and (A4) to Equation (A2),

(A5) $$S_{t_{e}}\left(t_{e}\right)=S_{t_{b}}(2000)*2^{(t_{e}-S_{t_{e}}\left(t_{e}\right)-2000)/d}$$

We seek a model that predicts survival, $S_{t_{e}}(\;)$, from end date, *t_e_*. However, Equation (A5) does not do that because $S_{t_{e}}\left(\;\right)$ appears on both sides of the “=” sign and thus picking a value for *t_e_* does not lead to Equation (A5) producing a survival prediction $S_{t_{e}}\left(t_{e}\right)$. We could consider solving Equation (A5) for $S_{t_{e}}\left(t_{e}\right)$ but this is a non-trivial task. The closest thing to a close-form solution would be an equation that includes the Lambert W-function (also known as the omega function, the product logarithm, and the product log function). However, an alternative is to solve Equation (A5) straightforwardly for end date *t_e_* instead:

$$t_{e}=\left[S_{t_{e}}\left(t_{e}\right)+d{log}_{2}\left(S_{t_{e}}\left(t_{e}\right)\right)\right]+k$$

with constant *k* defined by:

(A6) $$k=2000-d{log}_{2}\left(S_{t_{b}}\left(2000\right)\right)$$

Equation (A6), which is not exponential, readily supports finding *t_e_* given a value for $S_{t_{e}}\left(t_{e}\right)$, but what we need to do is the opposite and find survival time prediction $S_{t_{e}}\left(t_{e}\right)$ given *t_e_*. This may be achieved by testing different values for $S_{t_{e}}\left(t_{e}\right)$ to find one that produces the desired value of *t_e_* when plugged into Equation (A6). A systematic approach to determining what values of $S_{t_{e}}\left(t_{e}\right)$ to test in order to converge as closely as needed to the value that leads the target value of *t_e_* is provided by the well-known bisection method (Codesansar, 2024 [51]).

### Appendix A.2. Deriving a Death Year Survival Model from a Logistic Diagnosis Year Model

If a logistic model is chosen for how diagnosis year predicts survival time, transforming that into a model of how end year predicts survival time is similar to the process for the linear and exponential models.

The logistic equation in one of its general forms is

(A7) $$y=\frac{H}{1+e^{-r(x-x_{m})}}$$

where *H* is the maximum height at which this type of S-curve levels off, *r* represents how fast the curve rises, which is greatest at the *x*-axis location *x_m_*, where the height of the curve is *H*/2 (that is, the middle of the “S”), and *e* is typically Euler’s number although any other positive number could be used instead.

For the current domain we can restate Equation (A7) as

(A8) $$S_{t_{b}}\left(t_{b}\right)=\frac{H}{1+e^{-r(t_{b}-t_{H/2})}}$$

which transforms into the survival time vs. death year model, using the same process used above,

(A9) $$t_{e}=\frac{ln(s_{t_{e}}(t_{e}))}{r}-\frac{ln(H-s_{t_{e}}\left(t_{e}\right))}{r}+s_{t_{e}}(t_{e})+t_{H/2}$$

As for the exponential case, a bisection algorithm can be used with Equation (A9) to get $s_{t_{e}}\left(t_{e}\right)$ from *t_e_*.

### Appendix A.3. Discussion of the Exponential and Logistic Equations

Exponential modeling parallels linear modeling in several ways. Here, the trend curve describing how survival improves with diagnosis year is defined exponentially by Equation (A2) rather than linearly, and the parameters needing to be specified are the doubling time *d*, analogous to slope in the linear model, and like in the linear case, the *y*-axis intercept, which is $S_{t_{b}}\left(2000\right)$ in Equation (A2). Transforming Equation (A2) into a curve of survival time increase vs. death year gives Equation (A6). This is not exponential and indeed a model of increasing survival vs. death year cannot be exponential (Howell et al., 2019 [49]). Nevertheless, Equation (A6) can be fully specified by defining values for the same parameters as in Equation (A2), *d* and $S_{t_{b}}\left(2000\right)$.

Modeling using logistic curves follows the process for linear and exponential modeling. The trend curve for survival time vs. diagnosis year, Equation (A8), has parameters *r* and *t_H_*_/2_. Parameter *r* determines the maximum steepness or rate of increase of the S-curve, which occurs at the midpoint of the S where the variable *t_b_* = the parameter *t_H_*_/2_, and the height of the curve is half of its maximum height *H*. The value of *H* is 20 years in Figure 1 since the maximum possible survival time for an observation period of 20 years is 20 years. Parameter *t_H_*_/2_ is the time at which the height reaches *H*/2, and adjusting this parameter has the effect of shifting the S-curve left or right. Transformed into a model of survival time vs. death year, we obtain Equation (A9), which is not a logistic curve but has the same two parameters, whose values can be defined based on the data.

### Appendix A.4. Applying the Exponential Equations to Finding the Steepness Parameter of Figure 1

For the exponential model of Figure 1, to regress the steepness parameter in $S_{t_{e}}\left(t_{e}\right),$ we use Equation (A6) as follows.

- Set the value of $S_{t_{b}}\left(2000\right)$ in Equation (A6) using the equation shown in Figure 1 to find its prediction for survival in the year 2000. Solving $S_{t_{b}}\left(2000\right)=3.0748e^{0.0074*\left(x-1992\right)}$ yields $S_{t_{b}}\left(2000\right)=3.2623$.
- Substitute $S_{t_{b}}\left(2000\right)$ in Equation (A6) with 3.2623 to get
  (A10) $$t_{e}=\left[S_{t_{e}}\left(t_{e}\right)+d*{log}_{2}\left(S_{t_{e}}\left(t_{e}\right)\right)\right]+2000-d*{log}_{2}(3.262324)$$

- Regress *d* in Equation (A10) to the death date vs. survival time data to find the value for *d* that results in the equation fitting the data as well as possible. In doing so, for each candidate value of *d*, we need to compute $S_{t_{e}}\left(t_{e}\right)$ for each value of *t_e_* that we have data for. This will enable comparing the model curve $S_{t_{e}}\left(t_{e}\right)$ with each data item so that the fit of the curve can be calculated for that *d*. This regression process will identify the *d* with the best fit to the data. Calculating $S_{t_{e}}\left(t_{e}\right)$ was explained in the earlier discussion of Equation (A6).
